# A novel flexible endoscopic C-shaped suturing device for colonic wound closure in a porcine model

**DOI:** 10.1055/a-2860-0569

**Published:** 2026-05-13

**Authors:** Qiang Zhang, Zhou-yang Lian, Hong-yan Jin, Xiao-jun Ma, Jie Hu, Zhi Tang, Ran Song

**Affiliations:** 1 Guangdong Provincial Key Laboratory of Gastroenterology, Department of GastroenterologyNanfang Hospital, Southern Medical UniversityGuangzhouChina; 2 Department of RadiologyGuangdong Provincial People’s Hospital (Guangdong Academy of Medical Sciences), Southern Medical UniversityGuangzhouChina; 3 Micro-Tech (Nanjing) Co., Ltd, ChinaNanjingChina

**Fig. 1:**
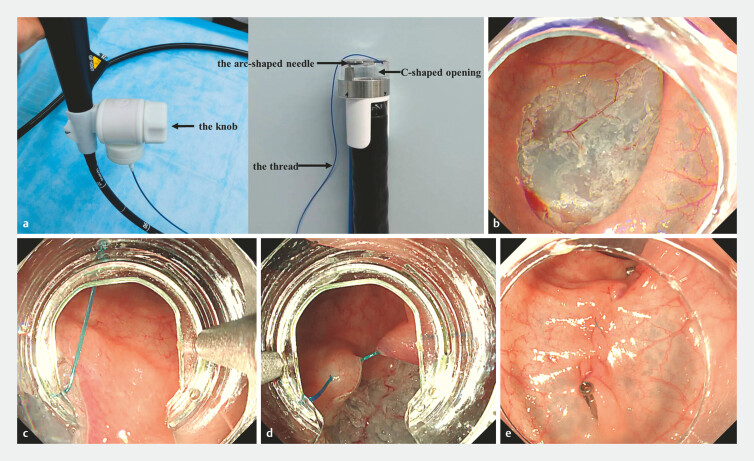
A novel endoscopic C-shaped suturing device (E-CSD) performing surgical thread-based suturing to close colonic wounds in a pig model. **a** The main structural features are an arc-shaped needle (constrained to a circular trajectory and attached to the suture thread), a long surgical thread, a delivery system (with an integrated knob for needle rotation), a 6-mm C-shaped opening, and a maximum outer diameter of 15.3 mm. **b** A 4 × 5 cm colonic wound in the colon 35 cm from the anus was created by endoscopic submucosal dissection. **c** The deviceʼs C-shaped opening was approximated to the tissue in the wound edge. **d** The needle tail-attached thread was passed through multiple points along the wound margin by rotating the knob to advance the arcuate needle 360°. **e** The wound was successfully closed following suture tensioning, securing, and cutting using the lock-and-cut device.

Suturing with surgical threads under flexible endoscopy effectively maintains wound closure without dehiscence until scar formation, promoting histological wound healing 1 . Conventional endoscopic hand-suturing (EHS) offers a simple, low-cost solution but it requires prolonged operative time 2 3 . We developed a novel endoscopic C-shaped suturing device (E-CSD; Micro-Tech [Nanjing] Co., Ltd, Nanjing, China) that performs suturing with surgical threads via 360° arcuate needle rotation. The device comprises ( Fig. 1 **a** ) an arcuate needle, a long surgical thread (a thread attached to the needle tail), a delivery system, an integrated locking-and-cutting device, and an optional grasper. Unlike EHS, the E-CSD uses semi-automatic operation to simplify the procedure and improve efficiency. Here, we applied the E-CSD to close a 4 × 5 cm colonic wound in the colon 35 cm from the anus created by endoscopic submucosal dissection in a 60-kg swine model ( Fig. 1 **b** ; Video 1 ). The E-CSD was mounted on the endoscope tip and advanced into the colon. The deviceʼs C-shaped opening was positioned against the wound edge ( Fig. 1 **c** ). When direct access was limited, the tissue was grasped with an endoscopic grasper and retracted into the opening. The operator rotated the delivery system knob to advance the arcuate needle 360°, passing the tail-attached thread through multiple points along the wound margin ( Fig. 1 **d** ). After the thread was passed along the wound edge, one thread end was secured with a suture-locking anchor and cut using the locking-and-cutting device; the opposite end was then tensioned, secured, and cut. The wound was successfully closed in 10 minutes ( Fig. 1 **e** ). During 1-month follow-up with weekly gastroscopy, no complications occurred, and the wound remained closed without dehiscence until scar healing. This case showed that the E-CSD achieved rapid and reliable wound closure with simplified operation in a porcine model. Further studies are warranted.

A colonic wound was successfully closed using the endoscopic C-shaped suturing device (E-CSD).Video 1

Endoscopy_UCTN_Code_TTT_1AQ_2AK
